# Emerging Role of Statin Therapy in Preventing Anthracycline‐Induced Cardiotoxicity

**DOI:** 10.1155/crp/9734869

**Published:** 2026-06-29

**Authors:** Elissar Mansour, Nawal Abi Raji, Pia Salloum, Nicolas Jreij, Thea Mila Ayoub, Mostafa Merheb, Philippe Attieh, Bernard Harbieh, Frederic Harb, Sami Azar, Hilda E. Ghadieh

**Affiliations:** ^1^ Department of Biomedical Sciences, Faculty of Medicine and Medical Sciences, University of Balamand, Al-Koura P.O. Box 100, Tripoli, Lebanon, balamand.edu.lb

**Keywords:** anthracyclines, cardioprotective, cardiotoxicity, chemotherapy, simvastatin, statins

## Abstract

Several randomized controlled trials (RCTs), animal studies, and observational studies have demonstrated the cardioprotective effects of statins, though their effectiveness varies. The observed variability may be attributed to individual differences, patient ages, disparities in statin type and dosage of anthracyclines (ANT) administered, and variations in cancer conditions among patients. Overall, statins play a beneficial role in reducing oxidative stress and inflammation, enhancing tumor sensitivity to chemotherapeutic drugs, improving mitochondrial function in cardiac cells, exerting antiapoptotic effects, and preserving left ventricular ejection fraction (LVEF). Despite these promising findings, the long‐term effects of statins remain unclear due to the lack of a standardized protocol. Statins may also cause side effects by depleting essential substances in the body. This limitation underscores the need for further research to assess their long‐term impact and establish standardized guidelines for dosing, duration, and potential side effects. The implications of this review highlight the importance of understanding the pleiotropic effects of statins to develop targeted therapies for chemotherapy‐induced cardiotoxicity. Additionally, integrating ongoing research into clinical guidelines is essential, ensuring that clinicians carefully consider patient‐specific factors when prescribing statins alongside ANT. This present review explores the potential role of statins in mitigating ANT‐induced cardiotoxicity, a major complication of chemotherapy that reduces LVEF and leads to heart failure (HF).

## 1. Introduction

Anthracyclines (ANT), a class of chemotherapeutic agents, widely used in the treatment for various types of cancers. ANT operate by targeting rapidly dividing cells and halting the spread of malignant tumors [1]. However, chemotherapy often comes with significant side effects. In the case of ANT, cardiotoxicity is a major complication, most characterized by reduced left ventricular ejection fraction (LVEF), potentially leading to heart failure (HF) and other cardiovascular diseases [2]. Cardiotoxicity is linked to various factors such as the dose of ANT received, pre‐existing cardiovascular illnesses, patient age, and concomitant therapies thus representing a major concern. It can impact the patient’s quality of life, even leading to premature death [3].

As a potential therapy to mitigate the cardiotoxic effect of ANT, statins showed anti‐inflammatory and antioxidant actions, thereby protecting the heart from damage caused by ANT [4]. They are also known as 3‐hydroxy‐3‐methylglutaryl coenzyme A (HMG‐CoA) reductase inhibitors, a class of cholesterol‐lowering drugs that have shown pleiotropic effects beyond their lipid‐lowering properties [5]. Several studies examined the use of statins in conjunction with anthracycline chemotherapy. While some studies reported a reduced incidence of cardiotoxicity with statin therapy, others failed to demonstrate significant benefit [2, 6–8]. Inconsistent findings highlight the need for further research to identify patient factors that can benefit from statin therapy.

This review aims to highlight the current knowledge about the cardioprotective effect of statins when administered with ANT chemotherapy. The possible mechanisms by which statins exert their cardioprotective impact along with the limitation in clinical data about this topic are discussed. Moreover, patient‐specific factors influencing the response to statin therapy, together with potential adverse effects and safety considerations, are discussed. Finally, ongoing research on this topic is to be highlighted in the hopes of providing a comprehensive understanding of the role of statins in protecting against cardiotoxicity from ANT chemotherapy and informing future research and clinical guidelines.

## 2. Materials and Methods

This review was designed as a clinically oriented narrative review. The aim is to establish the effects of incorporating statins into cancer treatment regimens, namely ANT, with the goal of preventing cardiotoxicity, with a rational synthesis of evidence.

To ensure a transparent and reproducible literature selection process, we used a search strategy that helped guide the narrative synthesis. A comprehensive search was conducted in PubMed and Google Scholar, for relevant articles related to the topic of statin use for cardioprotection in patients undergoing anthracycline chemotherapy. The following keywords and Boolean operators were used: (“statin” OR “lipid‐lowering drugs” OR “hydroxymethylglutaryl‐COA inhibitor” OR “simvastatin” OR “atorvastatin” OR “lovastatin” OR “rosuvastatin”) AND (“anthracyclines” OR “doxorubicin”) AND (“cancer” OR “breast cancer” OR “colon cancer”) AND (“cardiotoxicity” OR “heart failure” OR ”decreased left ventricular ejection fraction” OR “cardiomyopathy”). The search was limited to English‐language documents with visible abstracts. Notably, we included studies published in peer‐reviewed journals, retrospective clinical studies, observational studies, randomized controlled trials (RCTs), experimental studies, and meta‐analyses addressing the relationship between statins and risk of cardiotoxicity in anthracycline‐treated patients with long‐term effects. We excluded case reports, editorials, commentaries, comprehensive reviews, experimental preclinical studies without clear clinical implications or relation to the topic, nonpeer‐reviewed articles, and non‐English publications identified during the search process.

## 3. ANT and Their Cardiotoxic Effects: Mechanisms and Limitations

ANT, chemotherapeutic agents, known for their ability to target rapidly dividing cells and halt the spread of many malignant tumors [1]. For more than five decades, ANT have been used as cytostatic antibiotics. Since then, ANTs such as doxorubicin (DOX) and idarubicin have been prescribed in both children and adult patients with different forms of malignancies [9]. Even with new forms of treatment becoming available, ANT are still an integral part of breast cancer therapy because having an ANT‐based treatment has shown a 38% decrease in mortality in women with breast cancer [10]. However, there have been several limitations to its use due to many toxic side effects. ANT induce this toxic effect by generating reactive oxygen species (ROS) during intracellular metabolism and through the inhibition of topoisomerase IIβ (Top IIβ). The cardiac muscle cells are the most vulnerable due to their low levels of antioxidant enzymes, increased oxygen demand, and high mitochondrial content. Inhibiting topoisomerases, especially Top IIβ, leads to apoptotic pathways in cardiomyocytes being activated as well as impairment of mitochondrial growth and replication [10]. It is worth noting that mice with a cardiomyocyte‐specific deletion of Top IIβ did not exhibit symptoms related to anthracycline‐induced cardiotoxicity (AIC). On the other hand, ANT has been shown to increase ROS levels around 100‐fold. Elevated ROS causes irreversible damage to cell membranes and other cell structures through the oxidation of lipids, nucleic acids, and proteins [10]. Furthermore, DOX, a commonly used ANT, increases the levels of ROS by redox cycling a reaction catalyzed by nicotinamide adenine dinucleotide phosphate (NADPH) oxidase (NOX), and mitochondrial nicotinamide adenine dinucleotide + hydrogen (NADH) oxidase [11]. Also, an increase in nitric oxide (NO) by endothelial nitric oxide synthase (eNOS) and inducible nitric oxide synthase (iNOS) is noted, including peroxynitrite, a product of the reaction between superoxide and NO. Peroxynitrite can cause HF by inducing chronic or acute inflammation since cardiomyocytes are unable to adequately reduce the heightened oxidative stress [12].

**FIGURE 1:**
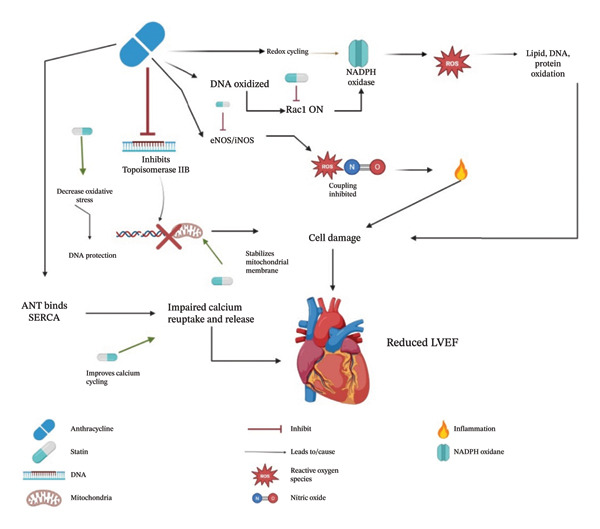
Pathophysiological cascade of anthracycline‐induced cardiomyocyte injury. This scheme illustrates the principal mechanisms underlying anthracycline‐induced cardiotoxicity. Anthracyclines promote redox cycling and Rac1‐mediated activation of NADPH, generating excess reactive oxygen species (ROS) and inducing the oxidation of protein, DNA, and lipid. The oxidative stress disrupts eNOS/iNOS coupling, further amplifying ROS production. The inhibition effect of anthracycline on topoisomerase IIβ leads to mitochondrial dysfunction and impaired genomic integrity. In addition, anthracyclines cause abnormal calcium handling and contractile dysfunction by binding to SERCA and impairing calcium reuptake. Collectively, these result in cardiomyocyte injury and hence a progressive reduction in left ventricular ejection fraction. Abbreviations: anthracycline (ANT), sarco/endoplasmic reticulum calcium ATPase (SERCA), left ventricular ejection fraction (LVEF), endothelial nitric oxide synthase (eNOS), inducible nitric oxide synthase (iNOS), nicotinamide adenine dinucleotide phosphate H+ oxidase (NOX), nitric oxide (NO), reactive oxygen species (ROS). Flame: inflammation, ROS: radicals, X: destruction, blue‐white pill: statin.

Ras‐homologous guanosine triphosphatases (Rho‐GTPases), cellular molecular switches, are activated by ANT. Ras‐related C3 botulinum toxin substrate 1 (Rac 1), a cofactor in the assembly of NOX subunits, is maintained in the active form since oxidized DNA bases caused by ANT administration can act as guanine nucleotide exchange factors (GEFs) promoting the accumulation of guanosine triphosphate (GTP)‐bound Rac 1. This prevents the cyclic change of GTP/guanosine diphosphate (GDP), increasing the level of ROS through NOX [13]. Finally, the cardiac muscles are highly dependent on the balance between calcium (Ca^2+^) release and reuptake at the level of the sarcoplasmic reticulum (SR). Disturbances caused by ANT can lead to cardiac dysfunction [14]. These mechanisms lead to the hallmark characteristic of AIC, which is reduced LVEF (Figure 1) [11].

## 4. The Cardioprotective and Antitumor Effects of Statins in ANT Therapy

Recent studies show the importance of statins, which are 3‐hydroxy‐3‐methylglutaryl‐CoA (HMG‐CoA) reductase inhibitors, in alleviating AIC [1, 15–17]. Despite being primarily used as lipid‐lowering agents, they are also known for their antioxidative and anti‐inflammatory effects, making them pleiotropic. Physicians often recommend statins to counteract the disruptive effects of ANT. Furthermore, statins can increase the sensitivity of certain tumors to chemotherapeutic agents, improving the efficacy of the treatment [18]. Statins have been associated with an enhancement in the LVEF in patients undergoing an ANT treatment. This improvement is mediated by a decrease in nitrotyrosine production in cardiac cells and the activation of mitochondrial‐located antioxidative and antiapoptotic mechanisms through the increase in basal cell‐leukemia/lymphoma‐2 (BCL‐2) expression, which in turn reduces the inflammatory response induced by ANT [19]. Mitochondrial superoxide dismutase (SOD2) expression has been shown to increase the following statin treatment. Findings propose that statins can also activate pathways that eliminate the ROS, rather than just decreasing their production [19]. In addition, they inhibit Rho‐GTPases [1]. This is done by preventing the synthesis of the mevalonate derivatives isoprenoids farnesyl pyrophosphate (FPP) and geranylgeranyl pyrophosphate (GGPP). Both derivatives take part in post‐translational prenylation as well as translocation of Rho GTPase members from the cytosol to the cell membrane [20].

Rho‐GTPase regulates endothelial eNOS and indirectly inhibits pro‐oxidative NADPH oxidase systems. Furthermore, in vitro studies have shown that DOX‐induced apoptosis is prevented in cardiomyocytes that were pretreated with atorvastatin [10, 21]. DOX treatment results in inhibiting single transducer and activator of transcription 3 (STAT3) from binding to Sp1 through activation of the forkhead box protein O1 (FOXO1) protein. The STAT3/Sp1 transcription complex is needed for the expression of the apoptotic inhibitor proteins. The FOXO1 protein is inactivated via protein phosphorylation when pretreated with a statin, allowing survivin to remain expressed in cardiomyocytes [10].

Simvastatin, like all other statins, when given with ANT, restores the mitochondria and induces an increase in the cytosolic Ca^2+^ storage levels [14]. DOX can directly bind to the sarco/endoplasmic reticulum calcium ATPase (SERCA2) and ryanodine receptor 2 (RyR2), altering the capacity to transfer calcium between the SR and the cytoplasm of muscle cells. Hence, they provide a cardioprotective effect by regulating Ca^2+^ cycling through SERCA2 independent of RyR2. This means that statins cannot prevent a defect in calcium release via the RyR2. Measuring the calcium transient duration (CaTD) after DOX‐statin injection in mice, where a significant decrease in CaTD is observed in statin‐DOX treatment compared to the control group only subjected to DOX [14].

Using western blotting, immunofluorescence, and cytofluorimetric analyses, investigators demonstrated that simvastatin significantly reduced mitochondrial connexin 43 (Cx43) overexpression induced by ANT [22]. Moreover, it increases the level of phosphorylated Cx43 in the cellular membranes. Cx43 is a protein from the connexin family, whereby the phosphorylated protein forms gap junctions and plays a pivotal role in cardioprotection through modulation of ROS signaling pathways. The phosphorylated form expressed in cardiomyocytes is ascribed to their ability to regulate the function of Rho‐GTPases [22]. Upon inhibition of Rho‐GTPases, NOX complex activity decreases, while eNOS activity is stabilized [13].

Furthermore, statins can reduce ANT‐induced inflammation by lowering inflammatory markers (C‐reactive protein [CRP], IL‐2, IL‐6, IL‐8, IL‐18, and tumor necrosis factor‐a [TNF‐a]) and downregulating the expression of toll‐like receptors (TLR4) on the surface of macrophages, neutrophils, and other immune cells [13]. Also, they inhibit the activity of nuclear factor‐kappa B (NF‐κB) through the blockage of RhoA/RhoB, which prevents the dissociation of IKb from NF‐κB. Hence, the downregulation of toll‐like receptor 4 (TLR4) and NF‐κB can reduce the expression of intercellular adhesion molecules (ICAMs) and chemotactic factors, thereby reducing inflammation. In addition, its anti‐apoptotic effect is induced by a decrease of cytochrome C release from the mitochondria [22]. Also, the administration of statins decreases the synthesis of farnesyl, an isoprenyl. Farnesyl is an important compound since it enables the anchoring of p21 to the cell membrane, enabling it to transduce a signal. P21 protein is a cyclin‐dependent kinase inhibitor that regulates the cell cycle. It is considered a tumor suppressor protein that alters the development of apoptosis. So, statins can impair the function of p21‐related signaling pathways, inducing apoptosis [23]. Interestingly, dose‐dependent effects have been reported, whereby higher statin doses enhance ANT‐mediated tumor cell killing, whereas lower doses may protect primary human endothelial cells from cell death. Aside from exhibiting a cardioprotective effect, they also help in protect intestinal tissues from the side effects induced by chemotherapy [24].

The imbalance between vasoconstriction and vasodilation through the inhibition of vascular endothelial growth factor (VEGF) production and its vascular endothelial growth factor receptor 2 (VEGFR2) caused by ANT is corrected by statins by stimulating the release of VEGF, increasing the synthesis and secretion of endothelin‐1 (ET‐1) [13]. Statins also exert anti‐inflammatory effects by decreasing the concentration of lipoprotein‐associated phospholipase (Lp‐PLA2) whose role is to hydrolyze low‐density lipoprotein (LDL)‐oxidized phospholipids that lead to vascular inflammation [13].

## 5. Clinical Evidence on the Effects of Statins

### 5.1. Reduction in Incidence of AIC

ANT are a major cause of cancer therapy‐related cardiac dysfunction (CTRCD), with the risk increasing with cumulative exposure and potentially resulting in HF or subclinical cardiac dysfunction that may manifest years after treatment [25]. Previous cardio‐protective techniques, including liposomal formulation, dexrazoxane, or neurohormonal blockers, aimed to reduce exposure to ANT but raised concern over compromising the anticancer effect of ANT and cancer recurrence. Other methods such as beta blockers, renin–angiotensin–aldosterone‐system (RAAS) blockers, and dexrazoxane were reserved for patients at high risk of developing CTRCD [20]. Statins, on the other hand, showed promising results in reducing cardiotoxicity risk and potentially lowering cancer mortality [4]. Several randomized controlled clinical trials (RCTs) compared the efficacy of statins to a control group (given either a placebo or no intervention), which showed a 54% relative risk reduction in cardiotoxicity incidence with statin use (risk ratio [RR]: 0.46; 95% confidence interval [CI]: 0.29 to 0.72; *p* < 0.001). Additionally, Felix et al. confirmed the safety of statins in cardioprotection, as no serious side effects, like liver enzyme rise and rhabdomyolysis, were reported compared to neurohormonal therapy, which can worsen hemodynamic effects in cancer patients vulnerable to diarrhea, vomiting, and dehydration [20]. Limitations to the study were also stated, such as the absence of a granular individual analysis, a heterogeneous definition of CTRCD, a small population pool, a study limit to high‐intensity statins, and absent representation of children and young adults, along with variability between the studies included, which did not majorly affect findings.

### 5.2. Reduction in Cardiotoxicity and Preservation in LVEF

Statins also seem to preserve LVEF in patients receiving ANT therapy. Though effective in treating various cancers, ANT and trastuzumab are both associated with cardiotoxicity, HF, and reduced LVEF. Cohort studies examined by Obasi et al. demonstrated better preservation of LVEF at baseline levels in patients on ANT and/or trastuzumab who were receiving statins compared to those who were not [weighted mean difference (WMD) = 6.14%, 95% CI (2.75–9.52), *p*  <  0.001] and RCTs [WMD = 6.25%, 95% CI (0.82–11.68), *p* = 0.024] [4]. Obasi et al. also provided limitations to the results, mentioning the small sample size, confounders unaccounted for, short follow‐up time of RCT patients, potential selection bias via omitted randomization information, and the need for further assessment of heterogeneity. Meta‐regression analysis showed a positive correlation between changes in LVEF levels and cumulative ANT dose, suggesting that statins could be more protective in patients with higher ANT exposure [25].

#### 5.2.1. HF Prevention

One irreversible and potentially lethal effect of cumulative doses of ANT is cardiomyopathy. The risk of cardiomyopathy increases with increased chemotherapy dose, but an observational study by Seicean et al. suggests that ongoing statin therapy, initiated before and continuing during chemotherapy, decreases the risk of HF over 2.2 ± 1.7 years of follow‐up [26]. However, the generalizability of these findings may be limited because the study population was derived from a single healthcare system.

#### 5.2.2. Further Clinical Evidence

Other potential direct and supportive roles of statins in cancer treatment are being studied, some suggesting that statins may enhance the accumulation of DOX in drug‐resistant tumor cells, leading to growth arrest and apoptosis while preserving normal cells [26]. Current research also supports the use of statins, describing them as safe with a potential to reduce recurrence and improve survival in certain cancers like breast cancer [4]. The pleiotropic effect exhibited by statins demonstrates their ability to provide cardioprotective effects against toxicity from therapies like ANT while reducing breast cancer incidence, recurrence, and mortality [27]. The antitumor effect of statins involves the suppression of the growth of breast cancer through multiple mechanisms, such as apoptosis, ferroptosis, and inhibition of enzymes or pathways required for tumor cells’ survival, such as Rho‐associated coiled‐coil containing protein kinase (ROCK) [27]. Lovastatin, for example, demonstrated reduced breast tumor growth and lung metastasis through a mitochondrial‐mediated, p53‐independent apoptotic mechanism in preclinical models. Furthermore, Simvastatin showed particular effectiveness against triple‐negative breast cancer cells by inhibiting HMG‐CoA reductase and modulating the mevalonate pathway [27]. Statins also may overcome chemotherapy resistance via modulation of ATP‐binding cassette subfamily B member 1 (ABCB1) transmembrane protein, enhanced pro‐apoptotic signaling (increased Bax, decreased Bcl‐2), and improving efficacy of agents like DOX, cyclophosphamide, insulin growth factor‐1 receptor (IGF‐1R) inhibitors, and human epidermal growth factor receptor 2 (HER2)‐targeted therapy [28]. Additionally, they may decrease local inflammation caused by radiation therapy postmastectomy, thus improving sensitivity of both stem cells and differentiated breast cancer cells to radiation therapy [27]. However, these findings are still limited to in vitro studies, where drug concentrations used are higher than in those typically achieved in humans [27]. Recent evidence suggests that statins may potentiate immunotherapy by downregulating programmed death ligand‐1 (PD‐L1) expression, upregulating CD8+ T‐cell‐mediated cytotoxicity, and promoting the conversion of immunologically “cold” tumors into more immunoreactive “hot” tumors, thereby supporting their use synergistically with breast cancer treatment [28]. On the other hand, statins also demonstrated a cardioprotective role in patients undergoing chemotherapy for breast cancer. For example, rosuvastatin (RSV) has a cardioprotective effect through reduction of oxidative DNA damage, myeloperoxidase (MPO) gene expression, a marker of inflammation and cardiotoxicity, and levels of IL‐6 and high sensitivity cardiac troponin I (Hs‐cTnI) in HER‐2 positive breast cancer patients undergoing chemotherapy [27]. In addition, protein convertase subtilisin/kexin type 9 (PCSK9) inhibitors (PCSK9i) (e.g., inclisiran and evolocumab) also showed the ability to mitigate the cardiotoxic effect of chemotherapy, likely via mechanisms independent of lipid lowering, as DOX enhances PCSK9 expression in cardiac tissues. Inclisiran improves cell viability, reduces apoptosis and cardiac damage biomarkers (heart‐type fatty acid binding protein [H‐FABP], troponin T, and brain natriuretic peptide [BNP]), lowers intracellular expression of inflammatory mediators involved in cardiotoxicity (NLRP3 and MyD88), and preserves mitochondrial and cytoskeletal integrity during DOX‐trastuzumab therapy [27]. Evolocumab similarly improves cardiac function and reduces myocardial damage and fibrosis induced by DOX by decreasing PCSK9 expression [27].

Cardio‐oncology guidelines recommend the use of statins for cardioprotection in patients at high‐risk of CTRCD [20]. This role as a preventive treatment for CTRCD has been tested in phase II RCTs and was attributed to the antioxidant and anti‐inflammatory properties of statins [25]. Nonetheless, these RCTs have produced conflicting results due to the small sample size, patient factors, and factors related to dosages of both statin therapy and chemotherapy, thus emphasizing the need for more large‐scale RCTs to clarify the impact of statins on cardiotoxicity prevention [4, 25].

## 6. Statin Therapy in Cancer Patients: Limited Clinical Data on Efficacy and Potential Risks

While statins show a promising cardioprotective effect, their efficacy varies based on patients′ factors, specific statins used, and chemotherapy regimens. A meta‐analysis that studied the effect of statins against CIC revealed a considerable decrease in chemotherapy‐induced cardiomyopathy (CIC) (WMD = −6.08%, 95% CI: −8.55 to −3.61, *p* < 0.001), along with a lesser decline in LVEF in statin‐treated patients compared to the control group (OR = 0.41, 95% CI = 0.28–0.60, *p* < 0.001). However, the short follow‐up period leaves the long‐term effect unclear [29]. Opposing views indicated the possible cardiotoxic effect of statins by increasing cardiac troponin levels. The study postulated that statins, through blocking cholesterol synthesis, can deplete the body of vital substances involved in cellular and mitochondrial function, particularly in cardiomyocytes [30].

Nevertheless, the benefits of statins far exceed these risks, with serious complications such as rhabdomyolysis and liver failure being rare and preventable with appropriate management [31]. Discontinuation of statin therapy due to intolerance would lead to exacerbation of cardiovascular symptoms, leading to atherosclerotic cardiovascular diseases [32]. Consequently, further research is warranted to investigate the mechanisms behind statin‐induced side effects along with identifying alternatives for patients who cannot tolerate the medications.

The lack of a standard protocol (including dosing, duration, time of initiation, and follow‐up) for the utilization of statins alongside chemotherapy against cardiotoxicity represents another crucial limitation [33]. In terms of dosage, high concentrations of statins appear to cause damage not only to cancer cells but also to neighboring normal cells, leading to myopathy and cardiac damage. However, it has been found that encapsulating the drug in liposomes reduces its amount and mitigates its harmful effects [33]. As for duration, pretreatment of mice with lovastatin for 1 week to 10 days has proven most beneficial to decrease levels of biochemical markers, while further trials are required to reach the appropriate lovastatin pretreatment schedule in humans receiving DOX [34]. Furthermore, Kim et al. deduced that the average follow‐up period for such studies lasts about 21.5 months, leaving long‐term preventive effects and the potential side effects of statins insufficiently characterized [29]. Evidence from an observational study by Seicean et al. suggests that statin use during ANT therapy is associated with a reduced incidence of HF over a follow‐up period of 2.55 ± 1.68, indicating a potential sustained cardioprotective effect beyond the acute phase [26].

Furthermore, meta‐analyses demonstrate that statins contribute to the preservation of LVEF and reduction in cardiotoxicity risk, supporting their role in mitigating long‐term cardiac dysfunction [4, 25].

Given these findings, future research trials should implement a more longitudinal study defining cardiovascular endpoints, including HF, cardiovascular mortality, and major adverse cardiovascular events, to evaluate the durability of statin‐mediated cardioprotection.

Long‐term monitoring of any cardiac‐related problems through the use of cardiac biomarkers, cardiac troponins, atrial natriuretic peptide (ANP), and BNP, is essential, as changes in these biomarkers may provide insight into ongoing myocardial injury and remodeling despite statin therapy [11]. Importantly, given the known association between ANT exposure and late‐onset cardiovascular disease, outcomes such as arrhythmias, ischemic heart disease, and overall cardiovascular mortality should also be evaluated in patients receiving in parallel statin therapy [3].

Accordingly, future trials evaluating statins’ cardioprotective effects should include more longitudinal follow‐up to determine any specific long‐term cardiovascular outcomes and possible side effects that should be anticipated in ANT‐treated patients supplemented with statins.

Furthermore, statin therapy may be associated with multisystem side effects besides its cardioprotective benefits and are summarized in Table 1.

**TABLE 1:** Side effects of statin therapy on multiple systems.

Side effect	Details
Musculoskeletal [35]	• Seen with all statin drugs, with myalgia being the most common form and rhabdomyolysis the most severe. • Rise in creatine kinase (CK). • Low rates of severe side effects caused by statins (0.1%–10%) • Exact mechanism of side effects unknown • Lowering the dose or total discontinuation of statin therapy depends on the severity of the musculoskeletal symptoms and cardiovascular risk.

Hepatic Dysfunction [35]	• A dose‐dependent rise in hepatic transaminase was observed in 1%–3% of patients on statin therapy. • No long‐term hepatic dysfunction was noticed, with very rare cases of statin‐related hepatic failure • No routine monitoring of liver function tests (LFTs) is recommended, and patients who experience mild changes in LFTs may safely continue statin therapy under observation.

Renal [35]	• Statins can cause mild proteinuria; there is no evidence of long‐term kidney dysfunction due to the therapy, and they seem well tolerated in chronic kidney disease (CKD) patients. • Therapy can be continued in patients with CKD with lower doses and avoidance of combination therapy (i.e., with fibrates or ezetimibe), except in cases of high cardiovascular risk.

Diabetes Mellitus [35]	• New onset of diabetes (NOD) was reported in patients on higher doses of statin therapy. • Some statins, such as pitavastatin did not affect blood glucose • Patients with diabetes are more likely to benefit from cardioprotective effects of statin therapy. • Routine blood glucose monitoring is recommended for diabetic patients on statins.

Malignancy [35]	• Few evidence report statins as risk factors for malignancies, while others report a protective role, stating that long‐term use of statins can reduce the risk of liver cancer, hematological cancers, and postmenopausal malignancy risk

Neurological [35]	• Some reports of statins’ association with mood symptoms and irritability. • No evidence of statin therapy affecting cognitive function. • Some studies reported a reduced risk of Alzheimer’s disease by statins [36]

Respiratory [35]	• Patients with chronic respiratory diseases can safely take statins.

Abbreviations: CK, creatine kinase; CKD, chronic kidney disease; LFTs, liver function tests; NOD, new onset of diabetes.

Although most studies endorse the use of statins for their cardioprotective effects against chemotherapy‐induced cardiotoxicity, all find common ground in noting that further research is required to clarify the underlying mechanism, optimal dosage, duration, and potential side effects.

## 7. Impact of Individual Variations on Statin Efficacy in Preventing AIC

Evidence from multiple studies suggests that the cardioprotective effects of statins against AIC may vary according to patient‐specific factors, including age, ANT type, dosage, pre‐existing cardiovascular conditions, and genetic predisposition.

### 7.1. Patient Age

Limited evidence is available regarding the influence of patients’ age on the efficacy of statins in preventing AIC. However, a meta‐analysis reported that participants in the included RCTs were aged 35–69 years, whereas those in the observational cohorts ranged from 40 to 70 years. No significant differences in statin efficacy were observed across these age ranges [4].

### 7.2. Type of Anthracycline

Several studies suggest that the cardioprotective efficacy of statins may vary according to the specific anthracycline used. In one study, atorvastatin (40 mg/day) administered to patients receiving DOX or idarubicin and without a conventional indication for statin therapy did not prevent declines in LVEF. Similarly, atorvastatin did not significantly affect circumferential strain measurements assessed 6 and 24 months after DOX treatment in patients with breast cancer or lymphoma [37]. In addition, lovastatin was effective in vivo when used against ANT‐induced subacute and subchronic cardiotoxicity brought about by DOX as well as the decline of diastolic cardiac function caused by DNA damage of cardiomyocytes leading to apoptosis [38]. As for RSV use against cardiotoxicity induced by DOX treatment in rats, it reduced the cardiotoxic events whether administered alone or combined with telmisartan, which can be detected via measuring the reduction in cardiac biomarkers [1].

As for other ANT, such as epirubicin, idarubicin, and mitoxantrone, cardiotoxicity is less pronounced than in conventional ANT. The former is cardiotoxic once used in higher amounts than the dosage of DOX, but it is a must if the same clinical effects are to be obtained [3]. Also, both idarubicin and mitoxantrone brought about less cardiotoxicity than DOX, as seen in preclinical studies and animal models [39, 40]. Therefore, because these agents generally exhibit lower cardiotoxicity than DOX, the relative cardioprotective benefit of statins may be less pronounced in patients receiving these anthracyclines [3].

### 7.3. Dose of ANT

Chronic treatment of mice with low‐dose DOX for 3 months resulted in increased expression of cardiomyocyte stress markers, including Interleukin‐6 (IL‐6), BNP, and heat shock protein beta‐1 (Hspa1b) RNA, which decreased following coadministration of lovastatin. Similar findings were observed in mice exposed to high‐dose ANT and lovastatin for 3 weeks, a model of subacute cardiotoxicity [41]. However, the protective effects of lovastatin were not observed in the chronic ANT exposure model. Therefore, the dosage and duration of ANT administration brought about overlapping statin mechanisms of anti‐inflammation and anti‐cardiotoxicity, whereby lovastatin was effective in treating early and part of the late DOX cardiac effects [41]. The varied statin effect against cardiotoxicity brought forth by dosage can also be seen in the statin to prevent the cardiotoxicity from anthracyclines (STOP‐CA) trial, further elaborated in Table 2. Differences in ANT dose may also explain variations in statin efficacy. In the STOP‐CA trial, patients received a cumulative ANT dose of 300 mg/m, compared with 240 mg/m^2^ in another RCT, and a cardioprotective benefit of statin therapy was observed in the former study [5].

**TABLE 2:** Proposed clinical approach for statin use in AIC, based on STOP‐CA [42].

Clinical question	Proposed approach	Evidence from STOP‐CA	Key caution
Who should be considered?	Consider atorvastatin 40 mg daily in adults with lymphoma receiving anthracyclines, especially higher‐risk patients.	Primary endpoint: significant LVEF decline occurred in 9% with atorvastatin vs. 22% with placebo	Evidence is strongest in lymphoma, not all cancer populations.
When should it start?	Start before the first anthracycline dose and continue during treatment; STOP‐CA used 12 months.	Atorvastatin was initiated before anthracycline exposure and continued for 12 months.	The trial does not establish whether later initiation provides the same benefit.
Who is most likely to benefit?	Favor use in patients at higher cardiotoxic risk rather than routine prophylaxis for everyone.	Greater signal in older patients, women, BMI ≥ 30, and those receiving cumulative anthracycline dose ≥ 250 mg/m^2^.	These subgroup findings are supportive but should still be interpreted cautiously
What benefit can be claimed?	Present statins as reducing imaging‐defined LV dysfunction during follow‐up.	STOP‐CA showed lower rates of clinically meaningful LVEF decline with atorvastatin.	No proven mortality benefit or definitive reduction in symptomatic heart failure.
Should it be routine for all patients?	No. A selective, risk‐adapted approach is more consistent with the current evidence.	The trial supports prophylaxis in a defined higher‐risk population rather than universal use.	Generalizability beyond lymphoma and lower‐risk groups remains limited.

*Note:* Atorvastatin 40 mg/day started before anthracyclines and continued for 12 months. STOP‐CA supports selective prophylactic atorvastatin in higher‐risk anthracycline‐treated adults with lymphoma, not universal use. Statin to prevent the cardiotoxicity from anthracyclines (STOP‐CA).

Abbreviations: BMI, body mass index; LVEF, left ventricular ejection fraction.

The STOP‐CA trial offers the strongest randomized evidence that statins protect the heart in patients who receive ANT. This double‐blind study involved 300 adults with lymphoma who were to be treated with ANT. Participants also received either atorvastatin 40 mg daily or a placebo. Prior to chemotherapy, the statin was started and given for 12 months. The primary outcome was a significant decline in left ventricular systolic function, defined as a drop of at least 10 percentage points to a final LVEF below 55%. This event occurred in 22% of the placebo group, compared to only 9% of the atorvastatin group. Also, a benefit was observed for the secondary endpoint of LVEF. Subgroup analyses showed a larger effect in patients who received higher doses of ANT and those with other risk factors. These results support a specific clinical strategy that reserves preventive statin use mainly for anthracycline‐treated patients who are at an increased risk of cardiotoxicity. The evidence is most solid for LVEF in lymphoma patients; moreover, the impact on overall HF and survival is still unclear.

### 7.4. Type of Statins

Lipophilic statins (atorvastatin, simvastatin) demonstrated a more tumor‐suppressive effect and lower cancer‐related mortality compared to hydrophilic statins (RSV, pravastatin), which showed lower bioavailability in peripheral tissues [2]. For example, atorvastatin administered prophylactically to lymphoma patients receiving ANT was associated with better preservation of LVEF [42]. Simvastatin, on the other hand, when encapsulated with DOX in a liposomal formulation, effectively reduced the risk of cardiotoxicity induced by free DOX and enhanced its anticancer effect [33]. Furthermore, pretreatment with lovastatin for a week also can protect from DOX‐induced cardiotoxicity and may represent a potentially less costly alternative to dexrazoxane and liposomal formulations, although further clinical validation is required [34].

Further analysis highlights the anti‐apoptotic effects of RSV and simvastatin against the chemotherapeutic agent cisplatin. Pretreatment with both statins was associated with improved echocardiographic parameters, lower heart rate, increased respiratory rate, and restoration of cardiac biomarker levels, with simvastatin showing slightly greater benefits than RSV [43].

### 7.5. Pre‐Existing Factors

Various risk factors (Table 3) could influence the extent of ANT toxicity and the efficiency of statins in providing cardioprotective efficacy. Important risk factors include ethnicity and age, with individuals of African American ethnicity and those older than 65 years or younger than 5 years exhibiting a higher risk of ANT‐related cardiotoxicity [3]. In addition, knowing that there is an association between cancer and cardiovascular risk factors, smoking, obesity (BMI > 30 kg/m^2^), and a sedentary lifestyle are also recognized risk factors.. As for alcohol, there are differing results as to whether it is helpful when in moderation or detrimental as a cardiovascular risk factor if exceedingly consumed [3]. Female sex, radiotherapy exposure, concomitant chemotherapy, Down syndrome, hereditary dilated cardiomyopathy, diabetes, and hypertension are associated with an increased risk of AIC and may identify patients who derive greater benefit from statin‐based cardioprotection [9].

**TABLE 3:** Pre‐existing risk factors associated with a higher risk of ANT toxicity affecting statins outcomes [3, 25].

Pre‐existing risk factors	Risk description
Ethnicity [3]	African American

Age [3]	> 65
	< 5

Cancer association [3]	Smoking
	BMI > 30

Alcohol [3]	Exceedingly consumed

Gender [9]	Female

Treatment [9]	Radiotherapy
	Chemotherapy

Congenital disorders [9]	Down syndrome
	Dilated cardiomyopathy

Health conditions [9]	Diabetes
	Hypertension

### 7.6. Genetic Predisposition

It was shown throughout one study that statins had varying effects when used against AIC while treating different types of cancer, including melanoma, brain cancer, solid tumors, breast, ovarian, prostate, colon, and lung cancer [2]. A few cell lines were fully susceptible to statins, others were partially susceptible, and some were not affected at all. Therefore, the cancer cells′ genetic background led to differing pharmacological effects of statins [2]. Further research is needed to clarify the role of genetic factors in determining the cardioprotective efficacy of statins during ANT therapy.

## 8. Comparative Efficacy

Statins have emerged as a potential prophylactic agent in preventing AIC, with numerous studies demonstrating their ability to mitigate cardiac dysfunction. Their cardioprotective effects are attributed to their anti‐inflammatory and antioxidant properties, which help combat oxidative stress and apoptosis in cardiomyocytes. In a meta‐analysis, statin use was associated with a significantly lower risk of cardiotoxicity in cohort studies (RR = 0.46, 95% CI: 0.27–0.78, *p* = 0.004) and a similar but nonsignificant trend in RCTs (RR = 0.49, 95% CI: 0.17–1.45, *p* = 0.20). Additionally, statins were shown to preserve LVEF, with a WMD of 6.14% in cohort studies and 6.25% in RCTs, indicating better cardiac function maintenance compared to non‐users [4, 44]. However, several limitations were noted, including heterogeneity in study design, small sample sizes, short follow‐up periods, nonrandomized study designs, poorly defined baseline characteristics, and inconsistent clinical endpoints. The mechanism behind this cardioprotection may involve the upregulation of mitochondrial SOD2 expression, as seen in preclinical studies where fluvastatin administration in mice exposed to DOX resulted in improved cardiac function and reduced inflammation [13, 19].

Despite these benefits, concerns have been raised regarding the metabolic effects of statins, particularly their potential to induce insulin resistance and glucose intolerance. While some studies report no impact on blood glucose levels, most indicate an increased risk of impaired glucose tolerance, elevated fasting insulin, and higher glycated hemoglobin (HbA1c), especially in patients with type 2 diabetes mellitus [13, 45]. The effects appear to be statin‐specific, as lipophilic atorvastatin was found to inhibit insulin‐mediated glucose uptake in cultured cardiomyocytes, whereas hydrophilic statins like pravastatin and RSV did not exhibit this effect. The underlying mechanism may involve suppression of ubiquinone synthesis, leading to decreased adenosine triphosphate (ATP) production necessary for insulin secretion, as well as interactions with peroxisome proliferator‐activated receptors, which regulate glucose metabolism and glucose transporter type 4 (GLUT‐4) expression. Interestingly, long‐term atorvastatin therapy in myocardial infarction patients was associated with increased ghrelin levels, a hormone that exerts cardioprotective effects and may counterbalance insulin resistance [13]. While these findings highlight the need for further research to clarify the metabolic risks associated with statin therapy, they also emphasize the importance of individualized treatment strategies to optfimize both cardioprotective and metabolic outcomes.

## 9. Balancing Benefits and Concerns of Statin Use in ANT Therapy

With all the great benefits statins can provide for patients under ANT therapy, there appear to be some potential concerns. The primary side effect is muscle pain, more common in immunocompromised patients and patients taking other drugs, which is perplexing when considering cancer patients on ANT like lymphomas, leukemias, and breast cancer. This remains a controversial topic because of the placebo effect as seen in a research study in which participant in the blinded phase reported more muscle symptoms than people who were aware of statins therapy [31]. Statin therapy has been associated with a spectrum of muscle‐related adverse effects ranging from myalgia to rhabdomyolysis. The increased utilization of lipid‐lowering medications has led to a rising incidence of rhabdomyolysis associated with HMG Co‐A inhibitors. Statin‐induced muscle toxicity, encompassing myopathy, myalgia, myositis, and rhabdomyolysis, stems from various pathophysiologic mechanisms [46]. Ceasing statin therapy effectively manages statin‐induced rhabdomyolysis, but continued elevation of creatine phosphokinase (CPK) levels post‐discontinuation warrants consideration for necrotizing autoimmune myopathy; therefore, concurrent administration of medications that augment muscle injury should be cautiously avoided [46]. Another potential side effect of the statin is abnormal liver function tests. Interestingly, elevations in liver enzymes depend on statin usage. Research comparing atorvastatin and RSV found that since atorvastatin is lipophilic, it is then metabolized by the liver [47].

## 10. Ongoing Research on Strategies for Monitoring Cardiotoxicity in Cancer Patients: Pharmacogenomic Models and Biomarkers

There is an urge to have strategies to monitor cardiotoxicity in cancer patients in which various tools are used, such as pharmacogenomic prediction models and biomarkers in which this assessment enhances an individualized approach [48]. Future strategies to ameliorate AIC can be reached by knowing the fine structural and functional properties of aldo‐keto reductase family 1 member A1 (AKR1A1) and CBR1, enabling the development of more selective oxidoreductase inhibitors [49]. Moreover, further clinical trials are needed to understand the mechanism of exercises while approaching the ANT treatment [9] as well as prove the effects of angiotensin‐converting enzyme inhibitor (ACEi) in cancer patients diagnosed with left ventricular dysfunction [50]. On top of that, further research is needed to determine the efficacy of this treatment by using it alone and combining it with other drugs [51]. This will help in better comprehending the pathogenesis of ANT treatment as well as in searching for better alternatives and will help in understanding new cardioprotective agents having low toxicity [52], besides defining its role as a prevention strategy in all patients and patients with high risk [53].

Additionally, upcoming research will help confirm or deny that the modulation of RAAS gene expression by anthracycline will lead to the activation of ACE/Ang II/angiotensin II receptor type 1 (AT‐1R) axis, in addition to a remarkable increase in plasma renin activity, angiotensin II levels, and upregulation of AT‐1R [54]. Confirming that by preventing AIC, patients can be protected from trastuzumab cardiotoxicity. This will need clinical trials with long observation as AIC has a late onset [54]. Besides determining the efficacy of administering renin angiotensin aldosterone system inhibitor (RAASi) after ANT‐based chemotherapy, it will confirm previous data showing that the dysregulation of RAAS is an important step in leading to cardiotoxicity [54].

This upcoming research should have a consistent trial design, as the usage of different endpoints and lack of consistent definition were the main limitations of the previous research [54]. In addition, safety measurements and treatment should be established to reach close monitoring required in the care of certain patients [54]. Professional organizations such as the American Heart Association (AHA) could contribute by developing standardized methodological recommendations and monitoring frameworks for future studies. In the meantime, researchers are awaiting the prevention of cardiac dysfunction during adjuvant breast cancer therapy (PRADAII) trial that is assessing the efficacy of an angiotensin receptor/neprilysin inhibitor (ARNi) in patients treated with ANT‐based chemotherapy for breast cancer [55]. Furthermore, collaboration between different specialties is highly encouraged for early identification and management of patients at risk, in which regular screening will further enhance the process [56].

## 11. Conclusion

Although ANT are highly effective chemotherapeutic agents, their clinical utility is constrained by the risk of cardiotoxicity, which can lead to reduced LVEF and subsequent cardiac dysfunction. Statins have shown promise in mitigating ANT‐induced cardiotoxicity through antioxidant, anti‐inflammatory, and other pleiotropic cardioprotective mechanisms. However, there are many individual variations in the benefits of statins, varying along the type of ANT used, its dosage, the statin type used, and pre‐existing cardiovascular and genetic factors present. Some studies have also shown side effects such as those on liver enzymes, rhabdomyolysis, and muscle pain. Therefore, further large‐scale studies with long‐term follow‐up are needed to better define the efficacy, safety profile, optimal use, and cardioprotective role of statins in patients receiving ANT therapy.

**Nomenclature**
AHA: American Heart Association
AKR1A1: Angiotensin‐converting enzyme inhibitor aldo‐keto reductase family 1 member A1
ACEi: Angiotensin‐converting enzyme inhibitor
ARNi: Angiotensin receptor/neprilysin inhibitor
AT‐1R: Angiotensin‐II receptor type 1
ANT: Anthracyclines
AIC: Anthracycline‐induced cardiotoxicity
ATP: Adenosine triphosphate
ABCB1: ATP‐binding cassette subfamily B member 1
ANP: Atrial natriuretic peptide
BCL‐2: Basal cell‐leukemia/lymphoma‐2
BNP: Brain natriuretic peptide
BMI: Body mass index
Ca^2+^
: Calcium
CaTD: Calcium transient duration
CTRCD: Cardiovascular dysfunction
ICAMS: Cellular adhesion molecules
CIC: Chemotherapy‐induced cardiomyopathy
CKD: Chronic kidney disease
CRP: C‐reactive protein
Cx43: Connexin 43
CPK: Creatine phosphokinase
DOX: Doxorubicin
ET‐1: Endothelin‐1
eNOS: Endothelial nitric oxide synthase
FPP: Isoprenoids farnesyl pyrophosphate
FOXO1: Forkhead box protein O1
GDP: Guanosine diphosphate
GGPP: Geranylgeranyl pyrophosphate
GLUT‐4: Glucose transporter type 4
GTP: Guanosine triphosphate
NADPH: Nicotinamide adenine dinucleotide phosphate
GEFs: Nucleotide exchange factor
HbA1c: Glycated hemoglobin
HF: Heart failure
Hspa1b: Heat shock protein b
HMG‐CoA: 3‐Hydroxy‐3‐methylglutaryl coenzyme A
HER2: Human epidermal growth factor receptor 2
Hs‐cTnI: High‐sensitivity cardiac troponin I
H‐FABP: Heart‐type fatty acid binding protein
iNOS: Inducible nitric oxide synthase
IL‐6: Interleukin‐6
LDL: Low‐density lipoprotein
LFTs: Liver function tests
LVEF: Left ventricular ejection fraction
Lp‐PLA2: Lipoprotein‐associated phospholipase
MPO: Myeloperoxidase
MyD88: Myeloid differentiation primary response 88
NF‐κB: Nuclear factor‐kappa B
NADH: Nicotinamide adenine dinucleotide + hydrogen
NOD: New onset of diabetes
NO: Nitric oxide
NOX: NADPH oxidase
NLRP3: Nod‐like receptor protein 3
PRADAII: Prevention of cardiac dysfunction during adjuvant breast cancer therapy
PD‐L1: Programmed death ligand‐1
PCSK9: Protein convertase subtilisin/kexin type 9
PCSK9i: Protein convertase subtilisin/kexin type 9 inhibitor
Rac 1: Ras‐related C3 botulinum toxin substrate 1
ROS: Reactive oxygen species
RAAS: Renin–angiotensin–aldosterone system
RCT: Randomized controlled clinical trial
Rho‐GTPases: Ras homologous guanosine triphosphatases
ROCK: Rho‐associated coiled‐coil containing protein kinase
RR: Risk ratio
RSV: Rosuvastatin
RyR2: Ryanodine receptor 2
SERCA2: Sarcoplasmic/endoplasmic reticulum Ca^2+^ transporting 2
STAT3: Single transducer and activator of transcription 3
STOP‐CA: Statin to prevent the cardiotoxicity from anthracyclines
SOD2: Superoxide dismutase
TLR4: Toll‐like receptor 4
TNF‐ α: Tumor necrosis factor‐alpha
Top IIβ: Topoisomerase IIβ
VEGF: Vascular endothelial growth factor
VEGFR2: Vascular endothelial growth factor receptor 2
WMD: Weighted mean difference

## Author Contributions

Conceptualization, Hilda E. Ghadieh; writing–original draft preparation, Elissar Mansour, Nawal Abi Raji, Pia Salloum, Nicolas Jreij, Thea Mila Ayoub, and Mostafa Merheb; writing–review and editing, Elissar Mansour, Nawal Abi Raji, Pia Salloum, Nicolas Jreij, Thea Mila Ayoub, Mostafa Merheb, Philippe Attieh, Bernard Harbieh, Frederic Harb, Sami Azar, and Hilda E. Ghadieh; supervision, Hilda E. Ghadieh; project administration, Hilda E. Ghadieh.

## Funding

This work received no specific funding.

## Disclosure

All authors have read and agreed to the published version of the manuscript.

## Ethics Statement

The authors have nothing to report.

## Consent

The authors have nothing to report.

## Conflicts of Interest

The authors declare no conflicts of interest.

## Data Availability

Data sharing is not applicable to this article as no new data were created or analyzed in this study.
